# Supplementary material for: Aedes koreicus, a vector on the rise: Pan-European genetic patterns, mitochondrial and draft genome sequencing
Source: PLoS One. 2022 Aug 1;17(8):e0269880. doi: 10.1371/journal.pone.0269880 (PMC9342712; doi:10.1371/journal.pone.0269880)
Supplement: S1 Table — A total of 130 COI sequences obtained from Aedes koreicus specimens collected in five European countries between 2008 and 2020 were analyzed. All the sequences are available in GenBank with the referred accession numbers. (PDF) [file pone.0269880.s001.pdf]

**S1 Table. Metadata of mosquito samples involved in the present study.** A total of 130 COI sequences obtained from *Aedes koreicus* specimens collected in five European countries between 2008 and 2020 were analyzed. All the sequences are available in GenBank with the referred accession numbers.

| Sample ID | COI GenBank Accession No | Haplotype | Country | Region | Province | Collection Place | Habitat | Coordinates | | Sample Collection Date |
|---|---|---|---|---|---|---|---|---|---|---|
| HU_Bar_2016_130 | OK668713 | Hap_3 | Hungary | Southern Transdanubia | Baranya | Pécs | urban | 46.139391 | 18.224344 | 2016.08.03. |
| HU_Bar_2016_162 | OK668736 | Hap_6 | Hungary | Southern Transdanubia | Baranya | Pécs | urban | 46.139391 | 18.224344 | 2016.08.24. |
| HU_Bar_2016_187 | OK668767 | Hap_16 | Hungary | Southern Transdanubia | Baranya | Pécs | urban | 46.139391 | 18.224344 | 2016.09.14. |
| HU_Bar_2017_214 | OK668715 | Hap_3 | Hungary | Southern Transdanubia | Baranya | Pécs | urban | 46.139391 | 18.224344 | 2017.05.31. |
| HU_Bar_2017_227 | OK668714 | Hap_3 | Hungary | Southern Transdanubia | Baranya | Pécs | urban | 46.139391 | 18.224344 | 2017.06.07. |
| HU_Bar_2017_241 | OK668730 | Hap_3 | Hungary | Southern Transdanubia | Baranya | Pécs | urban | 46.139391 | 18.224344 | 2017.06.14. |
| HU_Bar_2017_354 | OK668737 | Hap_6 | Hungary | Southern Transdanubia | Baranya | Pécs | urban | 46.139391 | 18.224344 | 2017.08.09. |
| HU_Bar_2017_371 | OK668816 | Hap_16 | Hungary | Southern Transdanubia | Baranya | Pécs | urban | 46.139391 | 18.224344 | 2017.08.16. |
| HU_Bar_2017_374 | OK668748 | Hap_3 | Hungary | Southern Transdanubia | Baranya | Pécs | urban | 46.139391 | 18.224344 | 2017.08.16. |
| HU_Bar_2017_378 | OK668764 | Hap_16 | Hungary | Southern Transdanubia | Baranya | Pécs | urban | 46.139391 | 18.224344 | 2017.08.16. |
| HU_Bar_2017_397 | OK668778 | Hap_12 | Hungary | Southern Transdanubia | Baranya | Pécs | urban | 46.139391 | 18.224344 | 2017.08.30. |
| HU_Bar_2018_430 | OK668834 | Hap_5 | Hungary | Southern Transdanubia | Baranya | Pécs | urban | 46.139391 | 18.224344 | 2018.05.16. |
| HU_Bar_2018_466 | OK668747 | Hap_6 | Hungary | Southern Transdanubia | Baranya | Pécs | urban | 46.139391 | 18.224344 | 2018.05.30. |
| HU_Bar_2018_596 | OK668712 | Hap_21 | Hungary | Southern Transdanubia | Baranya | Pécs | urban | 46.139391 | 18.224344 | 2018.06.27. |
| HU_Bar_2018_626 | OK668831 | Hap_7 | Hungary | Southern Transdanubia | Baranya | Pécs | urban | 46.139391 | 18.224344 | 2018.07.04. |
| HU_Bar_2018_660 | OK668828 | Hap_9 | Hungary | Southern Transdanubia | Baranya | Pécs | urban | 46.139391 | 18.224344 | 2018.07.11. |
| HU_Bar_2018_684 | OK668743 | Hap_6 | Hungary | Southern Transdanubia | Baranya | Pécs | urban | 46.139391 | 18.224344 | 2018.08.01. |
| HU_Bar_2018_697 | OK668819 | Hap_10 | Hungary | Southern Transdanubia | Baranya | Pécs | urban | 46.139391 | 18.224344 | 2018.08.15. |
| HU_Bar_2018_699 | OK668833 | Hap_31 | Hungary | Southern Transdanubia | Baranya | Pécs | urban | 46.139391 | 18.224344 | 2018.08.22. |
| HU_Bar_2018_708 | OK668709 | Hap_25 | Hungary | Southern Transdanubia | Baranya | Pécs | urban | 46.139391 | 18.224344 | 2018.08.29. |
| HU_Bar_2018_715 | OK668753 | Hap_3 | Hungary | Southern Transdanubia | Baranya | Pécs | urban | 46.139391 | 18.224344 | 2018.09.05. |
| HU_Bar_2019_740 | OK668750 | Hap_3 | Hungary | Southern Transdanubia | Baranya | Pécs | urban | 46.139391 | 18.224344 | 2019.05.15. |
| HU_Bar_2019_750 | OK668751 | Hap_3 | Hungary | Southern Transdanubia | Baranya | Pécs | urban | 46.139391 | 18.224344 | 2019.05.15. |
| HU_Bar_2019_753 | OK668795 | Hap_12 | Hungary | Southern Transdanubia | Baranya | Pécs | urban | 46.139391 | 18.224344 | 2019.05.15. |

| | | | | | | | | | | |
|---|---|---|---|---|---|---|---|---|---|---|
| HU_Bar_2019_788 | OK668822 | Hap_23 | Hungary | Southern Transdanubia | Baranya | Pécs | urban | 46.139391 | 18.224344 | 2019.06.05. |
| HU_Bar_2019_826 | OK668821 | Hap_24 | Hungary | Southern Transdanubia | Baranya | Pécs | urban | 46.139391 | 18.224344 | 2019.06.12. |
| HU_Bar_2019_912 | OK668749 | Hap_3 | Hungary | Southern Transdanubia | Baranya | Pécs | urban | 46.139391 | 18.224344 | 2019.07.03. |
| HU_Bar_2019_978 | OK668744 | Hap_6 | Hungary | Southern Transdanubia | Baranya | Pécs | urban | 46.139391 | 18.224344 | 2019.07.17. |
| HU_Bar_2019_1030 | OK668745 | Hap_6 | Hungary | Southern Transdanubia | Baranya | Pécs | urban | 46.139391 | 18.224344 | 2019.07.17. |
| HU_Bar_2019_1072 | OK668742 | Hap_6 | Hungary | Southern Transdanubia | Baranya | Pécs | urban | 46.139391 | 18.224344 | 2019.08.22. |
| HU_Bar_2019_1079 | OK668777 | Hap_12 | Hungary | Southern Transdanubia | Baranya | Pécs | urban | 46.139391 | 18.224344 | 2019.08.28. |
| HU_Bar_2019_1080 | OK668746 | Hap_6 | Hungary | Southern Transdanubia | Baranya | Pécs | urban | 46.139391 | 18.224344 | 2019.08.28. |
| HU_Bar_2019_1082 | OK668738 | Hap_6 | Hungary | Southern Transdanubia | Baranya | Pécs | urban | 46.139391 | 18.224344 | 2019.09.04. |
| HU_Bar_2019_1085 | OK668716 | Hap_3 | Hungary | Southern Transdanubia | Baranya | Pécs | urban | 46.139391 | 18.224344 | 2019.09.04. |
| HU_Bar_2019_1088 | OK668766 | Hap_16 | Hungary | Southern Transdanubia | Baranya | Pécs | urban | 46.139391 | 18.224344 | 2019.09.11. |
| HU_Bar_2019_1090 | OK668765 | Hap_16 | Hungary | Southern Transdanubia | Baranya | Pécs | urban | 46.139391 | 18.224344 | 2019.09.11. |
| HU_Bar_2020_2 | OK668717 | Hap_3 | Hungary | Southern Transdanubia | Baranya | Pécs | urban | 46.139391 | 18.224344 | 2020.08.27. |
| HU_Bar_2020_3 | OK668763 | Hap_16 | Hungary | Southern Transdanubia | Baranya | Pécs | urban | 46.139391 | 18.224344 | 2020.09.11. |
| HU_Bud_2020_1 | OK668752 | Hap_3 | Hungary | Central-Hungary | Pest | Budapest | urban | 47.519286 | 19.077579 | 2020.09.29. |
| HU_Bud_2020_2 | OK668710 | Hap_22 | Hungary | Central-Hungary | Pest | Budapest | urban | 47.519286 | 19.077579 | 2020.09.29. |
| HU_Bud_2020_4 | OK668711 | Hap_27 | Hungary | Central-Hungary | Pest | Budapest | urban | 47.519286 | 19.077579 | 2020.09.29. |
| SN_Dra_2013_539 | OK668820 | Hap_20 | Slovenia | Drava | | Lovrenc na Dravskem polju | urban | 46.373849 | 15.783902 | 2013.09.01. |
| SN_Dra_2013_540 | OK668759 | Hap_3 | Slovenia | Drava | | Lovrenc na Dravskem polju | urban | 46.373849 | 15.783902 | 2013.09.01. |
| SN_Vip_2019_01 | OK668835 | Hap_4 | Slovenia | Goriška | Vipava valley | Črniče | cemetery | 45.905009 | 13.777402 | 2019.09.04. |
| IT_Ven_2020_1 | OK668785 | Hap_12 | Italy | Veneto | Padova | Baone | urban | 45.249579 | 11.673661 | 2019.09.27. |
| IT_Ven_2020_2 | OK668720 | Hap_3 | Italy | Veneto | Belluno | Pedavena | urban | 46.051107 | 11.872342 | 2020.05.14. |
| IT_Ven_2020_3 | OK668779 | Hap_12 | Italy | Veneto | Vicenza | Valli del Pasubio | urban | 45.765623 | 11.238465 | 2020.05.14. |
| IT_Ven_2020_4 | OK668829 | Hap_29 | Italy | Veneto | Treviso | Vittorio Veneto | urban | 46.081524 | 12.336247 | 2020.06.07. |
| IT_Fri_2020_5 | OK668719 | Hap_3 | Italy | Friuli Venzia Giulia | Pordenone | Maniago | urban | 46.164100 | 12.683844 | 2020.06.19. |
| IT_Fri_2020_6 | OK668727 | Hap_3 | Italy | Friuli Venzia Giulia | Pordenone | Arba | urban | 46.143553 | 12.785998 | 2020.06.19. |
| IT_Ven_2020_8 | OK668731 | Hap_3 | Italy | Veneto | Verona | Monteforte d'Alpone | urban | 45.444958 | 11.285270 | 2020.06.30. |
| IT_Tre_2019_2 | OK668790 | Hap_12 | Italy | Trentino-Alto Adige | Trento | Grigno | urban | 46.019535 | 11.631182 | 2019.10.05. |
| IT_Tre_2019_4 | OK668789 | Hap_12 | Italy | Trentino-Alto Adige | Trento | Grigno | urban | 46.019535 | 11.631182 | 2019.10.05. |
| IT_Tre_2019_5 | OK668784 | Hap_12 | Italy | Trentino-Alto Adige | Trento | Grigno | urban | 46.019535 | 11.631182 | 2019.10.05. |

| | | | | | | | | | | |
|---|---|---|---|---|---|---|---|---|---|---|
| IT_Tre_2019_6 | OK668758 | Hap_19 | Italy | Trentino-Alto Adige | Trento | Grigno | urban | 46.019535 | 11.631182 | 2019.10.05. |
| IT_Tre_2019_8 | OK668739 | Hap_6 | Italy | Trentino-Alto Adige | Trento | Grigno | urban | 46.019535 | 11.631182 | 2019.10.05. |
| IT_Tre_2019_11 | OK668794 | Hap_17 | Italy | Trentino-Alto Adige | Trento | Grigno | urban | 46.019535 | 11.631182 | 2019.10.05. |
| IT_Tre_2019_14 | OK668741 | Hap_6 | Italy | Trentino-Alto Adige | Trento | Grigno | urban | 46.019535 | 11.631182 | 2019.10.05. |
| IT_Tre_2019_16 | OK668740 | Hap_6 | Italy | Trentino-Alto Adige | Trento | Grigno | urban | 46.019535 | 11.631182 | 2019.10.05. |
| IT_Tre_2019_18 | OK668787 | Hap_12 | Italy | Trentino-Alto Adige | Trento | Grigno | urban | 46.019535 | 11.631182 | 2019.10.05. |
| IT_Tre_2019_19 | OK668788 | Hap_12 | Italy | Trentino-Alto Adige | Trento | Grigno | urban | 46.019535 | 11.631182 | 2019.10.05. |
| IT_Tre_2019_20 | OK668726 | Hap_3 | Italy | Trentino-Alto Adige | Trento | Grigno | urban | 46.019535 | 11.631182 | 2019.10.05. |
| IT_Tre_2020_21 | OK668826 | Hap_30 | Italy | Trentino-Alto Adige | Trento | Castel Ivano | urban | 46.072517 | 11.518469 | 2020.05.26. |
| IT_Tre_2020_22 | OK668757 | Hap_18 | Italy | Trentino-Alto Adige | Trento | Castel Ivano | urban | 46.072517 | 11.518469 | 2020.05.26. |
| IT_Tre_2020_26 | OK668800 | Hap_12 | Italy | Trentino-Alto Adige | Trento | Castel Ivano | urban | 46.072517 | 11.518469 | 2020.05.26. |
| IT_Tre_2020_29 | OK668825 | Hap_3 | Italy | Trentino-Alto Adige | Trento | Castel Ivano | urban | 46.072517 | 11.518469 | 2020.05.26. |
| IT_Tre_2020_30 | OK668786 | Hap_12 | Italy | Trentino-Alto Adige | Trento | Castel Ivano | urban | 46.072517 | 11.518469 | 2020.05.26. |
| IT_Tre_2020_32 | OK668791 | Hap_12 | Italy | Trentino-Alto Adige | Trento | Castel Ivano | urban | 46.072517 | 11.518469 | 2020.05.26. |
| IT_Tre_2020_35 | OK668796 | Hap_12 | Italy | Trentino-Alto Adige | Trento | Castel Ivano | urban | 46.072517 | 11.518469 | 2020.05.26. |
| IT_Tre_2020_41 | OK668725 | Hap_3 | Italy | Trentino-Alto Adige | Trento | San Michele all'Adige | urban | 46.193126 | 11.135434 | 2020.05.26. |
| IT_Tre_2020_44 | OK668760 | Hap_3 | Italy | Trentino-Alto Adige | Trento | San Michele all'Adige | urban | 46.193126 | 11.135434 | 2020.05.26. |
| IT_Tre_2020_45 | OK668815 | Hap_12 | Italy | Trentino-Alto Adige | Trento | San Michele all'Adige | urban | 46.193126 | 11.135434 | 2020.05.26. |
| IT_Tre_2020_52 | OK668781 | Hap_12 | Italy | Trentino-Alto Adige | Trento | San Michele all'Adige | urban | 46.193126 | 11.135434 | 2020.05.26. |
| IT_Tre_2020_53 | OK668783 | Hap_12 | Italy | Trentino-Alto Adige | Trento | San Michele all'Adige | urban | 46.193126 | 11.135434 | 2020.05.26. |
| IT_Tre_2020_54 | OK668793 | Hap_12 | Italy | Trentino-Alto Adige | Trento | San Michele all'Adige | urban | 46.193126 | 11.135434 | 2020.05.26. |
| IT_Tre_2020_55 | OK668797 | Hap_12 | Italy | Trentino-Alto Adige | Trento | San Michele all'Adige | urban | 46.193126 | 11.135434 | 2020.05.26. |
| IT_Tre_2020_56 | OK668801 | Hap_12 | Italy | Trentino-Alto Adige | Trento | San Michele all'Adige | urban | 46.193126 | 11.135434 | 2020.05.26. |
| IT_Tre_2020_57 | OK668782 | Hap_12 | Italy | Trentino-Alto Adige | Trento | San Michele all'Adige | urban | 46.193126 | 11.135434 | 2020.05.26. |
| IT_Tre_2020_58 | OK668780 | Hap_12 | Italy | Trentino-Alto Adige | Trento | San Michele all'Adige | urban | 46.193126 | 11.135434 | 2020.05.26. |
| IT_Tre_2020_59 | OK668798 | Hap_12 | Italy | Trentino-Alto Adige | Trento | San Michele all'Adige | urban | 46.193126 | 11.135434 | 2020.05.26. |
| IT_Tre_2020_60 | OK668802 | Hap_12 | Italy | Trentino-Alto Adige | Trento | San Michele all'Adige | urban | 46.193126 | 11.135434 | 2020.05.26. |
| IT_Tre_2020_61 | OK668827 | Hap_12 | Italy | Trentino-Alto Adige | Trento | San Michele all'Adige | urban | 46.193126 | 11.135434 | 2020.05.26. |
| IT_Tre_2020_62 | OK668803 | Hap_12 | Italy | Trentino-Alto Adige | Trento | San Michele all'Adige | urban | 46.193126 | 11.135434 | 2020.05.26. |

| | | | | | | | | | | |
|---|---|---|---|---|---|---|---|---|---|---|
| IT_Tre_2020_63 | OK668804 | Hap_12 | Italy | Trentino-Alto Adige | Trento | San Michele all'Adige | urban | 46.193126 | 11.135434 | 2020.05.26. |
| IT_Tre_2020_64 | OK668814 | Hap_12 | Italy | Trentino-Alto Adige | Trento | San Michele all'Adige | urban | 46.193126 | 11.135434 | 2020.05.26. |
| IT_Tre_2020_65 | OK668799 | Hap_12 | Italy | Trentino-Alto Adige | Trento | San Michele all'Adige | urban | 46.193126 | 11.135434 | 2020.05.26. |
| IT_Tre_2020_66 | OK668792 | Hap_12 | Italy | Trentino-Alto Adige | Trento | San Michele all'Adige | urban | 46.193126 | 11.135434 | 2020.05.26. |
| IT_Tre_2020_68 | OK668761 | Hap_3 | Italy | Trentino-Alto Adige | Trento | Villamontagna | urban | 46.089603 | 11.159079 | 2020.05.26. |
| IT_Tre_2020_71 | OK668754 | Hap_3 | Italy | Trentino-Alto Adige | Trento | Villamontagna | urban | 46.089603 | 11.159079 | 2020.05.26. |
| IT_Tre_2020_72 | OK668755 | Hap_3 | Italy | Trentino-Alto Adige | Trento | Villamontagna | urban | 46.089603 | 11.159079 | 2020.05.26. |
| IT_Tre_2020_73 | OK668824 | Hap_3 | Italy | Trentino-Alto Adige | Trento | Villamontagna | urban | 46.089603 | 11.159079 | 2020.05.26. |
| IT_Tre_2020_74 | OK668823 | Hap_26 | Italy | Trentino-Alto Adige | Trento | Villamontagna | urban | 46.089603 | 11.159079 | 2020.05.26. |
| IT_Tre_2020_75 | OK668836 | Hap_3 | Italy | Trentino-Alto Adige | Trento | Villamontagna | urban | 46.089603 | 11.159079 | 2020.05.26. |
| IT_Tre_2020_76 | OK668837 | Hap_2 | Italy | Trentino-Alto Adige | Trento | Villamontagna | urban | 46.089603 | 11.159079 | 2020.05.26. |
| IT_Tre_2020_77 | OK668724 | Hap_3 | Italy | Trentino-Alto Adige | Trento | Villamontagna | urban | 46.089603 | 11.159079 | 2020.05.26. |
| IT_Tre_2020_78 | OK668729 | Hap_3 | Italy | Trentino-Alto Adige | Trento | Villamontagna | urban | 46.089603 | 11.159079 | 2020.05.26. |
| IT_Tre_2020_79 | OK668718 | Hap_3 | Italy | Trentino-Alto Adige | Trento | Villamontagna | urban | 46.089603 | 11.159079 | 2020.05.26. |
| IT_Tre_2020_81 | OK668728 | Hap_3 | Italy | Trentino-Alto Adige | Trento | Villamontagna | urban | 46.089603 | 11.159079 | 2020.05.26. |
| DE_Hes_2019_1 | OK668772 | Hap_11 | Germany | Hessen | Wiesbaden | Wiesbaden | cemetery | 50.05853 | 8.268892 | 2019.08.08. |
| DE_Hes_2019_2 | OK668776 | Hap_11 | Germany | Hessen | Wiesbaden | Wiesbaden | cemetery | 50.05853 | 8.268892 | 2019.08.08. |
| DE_Hes_2019_3 | OK668805 | Hap_15 | Germany | Hessen | Wiesbaden | Wiesbaden | cemetery | 50.05853 | 8.268892 | 2019.08.29. |
| DE_Hes_2019_4 | OK668775 | Hap_11 | Germany | Hessen | Wiesbaden | Wiesbaden | cemetery | 50.05853 | 8.268892 | 2019.08.29. |
| DE_Hes_2019_5 | OK668773 | Hap_11 | Germany | Hessen | Wiesbaden | Wiesbaden | cemetery | 50.05853 | 8.268892 | 2019.08.29. |
| DE_Hes_2019_6 | OK668769 | Hap_11 | Germany | Hessen | Wiesbaden | Wiesbaden | cemetery | 50.05853 | 8.268892 | 2019.08.29. |
| DE_Hes_2019_7 | OK668770 | Hap_11 | Germany | Hessen | Wiesbaden | Wiesbaden | cemetery | 50.05853 | 8.268892 | 2019.08.29. |
| DE_Hes_2019_8 | OK668830 | Hap_8 | Germany | Hessen | Wiesbaden | Wiesbaden | cemetery | 50.05853 | 8.268892 | 2019.08.29. |
| DE_Hes_2019_9 | OK668806 | Hap_15 | Germany | Hessen | Wiesbaden | Wiesbaden | cemetery | 50.05853 | 8.268892 | 2019.08.29. |
| DE_Hes_2019_10 | OK668771 | Hap_11 | Germany | Hessen | Wiesbaden | Wiesbaden | cemetery | 50.05853 | 8.268892 | 2019.08.29. |
| DE_Hes_2019_11 | OK668774 | Hap_11 | Germany | Hessen | Wiesbaden | Wiesbaden | cemetery | 50.05853 | 8.268892 | 2019.08.29. |
| DE_Hes_2019_12 | OK668768 | Hap_11 | Germany | Hessen | Wiesbaden | Wiesbaden | cemetery | 50.05853 | 8.268892 | 2019.08.29. |
| DE_Hes_2019_13 | OK668817 | Hap_11 | Germany | Hessen | Wiesbaden | Wiesbaden | cemetery | 50.015663 | 8.281782 | 2019.08.15. |
| DE_Hes_2019_26 | OK668818 | Hap_11 | Germany | Hessen | Wiesbaden | Wiesbaden | cemetery | 50.097884 | 8.270688 | 2019.09.27. |

| | | | | | | | | | | |
|---|---|---|---|---|---|---|---|---|---|---|
| DE_Hes_2019_27 | OK668808 | Hap_13 | Germany | Hessen | Wiesbaden | Wiesbaden | cemetery | 50.097884 | 8.270688 | 2019.09.27. |
| DE_Hes_2019_28 | OK668807 | Hap_14 | Germany | Hessen | Wiesbaden | Wiesbaden | cemetery | 50.097884 | 8.270688 | 2019.09.27. |
| DE_Hes_2019_29 | OK668812 | Hap_11 | Germany | Hessen | Wiesbaden | Wiesbaden | cemetery | 50.097884 | 8.270688 | 2019.09.27. |
| DE_Hes_2019_30 | OK668810 | Hap_11 | Germany | Hessen | Wiesbaden | Wiesbaden | cemetery | 50.097884 | 8.270688 | 2019.09.27. |
| DE_Hes_2019_31 | OK668811 | Hap_11 | Germany | Hessen | Wiesbaden | Wiesbaden | cemetery | 50.097884 | 8.270688 | 2019.09.27. |
| DE_Hes_2019_32 | OK668809 | Hap_11 | Germany | Hessen | Wiesbaden | Wiesbaden | cemetery | 50.076252 | 8.181752 | 2019.08.29. |
| DE_Hes_2019_33 | OK668813 | Hap_28 | Germany | Hessen | Wiesbaden | Wiesbaden | cemetery | 50.076252 | 8.181752 | 2019.08.29. |
| BE_Fla_2018_1 | OK668734 | Hap_6 | Belgium | Flandria | Limburg | Maasmechelen | industrial area | 50.995194 | 5.621833 | 2018.07.03. |
| BE_Fla_2018_2 | OK668723 | Hap_3 | Belgium | Flandria | Limburg | Maasmechelen | industrial area | 50.995194 | 5.621833 | 2018.07.03. |
| BE_Fla_2018_3 | OK668756 | Hap_6 | Belgium | Flandria | Limburg | Maasmechelen | industrial area | 50.995194 | 5.621833 | 2018.07.03. |
| BE_Fla_2018_4 | OK668732 | Hap_6 | Belgium | Flandria | Limburg | Maasmechelen | industrial area | 50.995194 | 5.621833 | 2018.07.17. |
| BE_Fla_2018_5 | OK668762 | Hap_3 | Belgium | Flandria | Limburg | Maasmechelen | industrial area | 50.995194 | 5.621833 | 2018.07.17. |
| BE_Fla_2018_6 | OK668832 | Hap_6 | Belgium | Flandria | Limburg | Maasmechelen | industrial area | 50.995194 | 5.621833 | 2018.07.17. |
| BE_Fla_2018_7 | OK668733 | Hap_6 | Belgium | Flandria | Limburg | Maasmechelen | industrial area | 50.995194 | 5.621833 | 2018.06.19. |
| BE_Fla_2018_8 | OK668735 | Hap_6 | Belgium | Flandria | Limburg | Maasmechelen | industrial area | 50.995194 | 5.621833 | 2018.06.19. |
| BE_Fla_2018_9 | OK668722 | Hap_3 | Belgium | Flandria | Limburg | Maasmechelen | industrial area | 50.995194 | 5.621833 | 2018.06.19. |
| BE_Fla_2018_10 | OK668721 | Hap_3 | Belgium | Flandria | Limburg | Maasmechelen | industrial area | 50.995194 | 5.621833 | 2018.06.19. |
| GenBank | JF430393 | Hap_1 | Belgium | Flandria | Limburg | Maasmechelen | industrial area | 50.996261 | 5.619860 | 2008.05.27. |
