# Supplementary material for: Aedes koreicus, a vector on the rise: Pan-European genetic patterns, mitochondrial and draft genome sequencing
Source: PLoS One. 2022 Aug 1;17(8):e0269880. doi: 10.1371/journal.pone.0269880 (PMC9342712; doi:10.1371/journal.pone.0269880)
Supplement: S2 Table — The completeness of the genome assembly was evaluated with the Benchmarking Universal Single-Copy Orthologs (BUSCO, v 4.1.2) software, using the Diptera lineage dataset. C: complete; S: single; D: duplicated; F: fragment; M: missing. (PDF) [file pone.0269880.s002.pdf]

S2 Table. Quality features of the complete genome sequence of *Aedes koreicus* achieved by Oxford Nanopore and Illumina sequencing. The completeness of the genome assembly was evaluated with the Benchmarking Universal Single-Copy Orthologs (BUSCO, v 4.1.2) software, using the Diptera lineage dataset. C: complete; S: single; D: duplicated; F: fragment; M: missing.

**Sequencing platform**	**Assemblies**	**BUSCO analysis numbers**				
		C	S	D	F	M
Nanopore	mosq.contigs.fasta	1,169	1,136	33	659	1457
Nanopore+Illumina	pilon (1st iteration)	2,384	2,325	59	299	602
Nanopore+Illumina	pilon (3rd iteration)	2,448	2,380	68	270	567
		**BUSCO analysis percentages**				
		C (%)	S (%)	D (%)	F (%)	M (%)
Nanopore	mosq.contigs.fasta	35.59	34.58	1.00	20.06	44.35
Nanopore+Illumina	pilon (1st iteration)	72.57	70.78	1.80	9.10	18.33
Nanopore+Illumina	pilon (3rd iteration)	74.52	72.45	2.07	8.22	17.26
