# Supplementary material for: Aedes koreicus, a vector on the rise: Pan-European genetic patterns, mitochondrial and draft genome sequencing
Source: PLoS One. 2022 Aug 1;17(8):e0269880. doi: 10.1371/journal.pone.0269880 (PMC9342712; doi:10.1371/journal.pone.0269880)
Supplement: S1 Appendix — (PDF) [file pone.0269880.s003.pdf]

S1 Appendix. Results of Tajima's D, Fu and Li's Neutrality Tests calculations.

Sequences	Tajima's D	P value	Fu and Li's		P value	
			D test	F test	D test	F test
Full dataset	-2.10058	< 0.05	-6.26734	-5.43285	< 0.02	< 0.02
Hungary	-1.46929	P > 0.10	-2.56505	-2.59537	< 0.05	< 0.05
Belgium	-0.83418	> 0.10	-1.63423	-1.73384	> 0.10	> 0.10
Italy	-1.83949	P < 0.05	-3.92005	-3.77266	< 0.02	< 0.02
Germany	-1.39905	> 0.10	-1.04259	-1.33169	> 0.10	> 0.10
