# Supplementary material for: Aedes koreicus, a vector on the rise: Pan-European genetic patterns, mitochondrial and draft genome sequencing
Source: PLoS One. 2022 Aug 1;17(8):e0269880. doi: 10.1371/journal.pone.0269880 (PMC9342712; doi:10.1371/journal.pone.0269880)
Supplement: S2 Appendix — “Freq” indicates the number of samples belong to the given haplotype, name of the “Sequences” refers to the GenBank Accession Numbers listed in the S1 Table. (PDF) [file pone.0269880.s004.pdf]

S2 Appendix. Distribution of the 130 investigated *Aedes koreicus* samples among the 31 different haplotypes (Hap#). "Freq" indicates the number of samples belong to the given haplotype, name of the "Sequences" refers to the GenBank Accession Numbers listed in the S1 Table.

[Hap# Freq. Sequences]
[Hap_1: 1   JF430393_1]
[Hap_2: 1   OK668837_1]
[Hap_3: 34   OK668836_1 OK668760_1 OK668751_1 OK668750_1 OK668749_1 OK668759_1 OK668729_1 OK668728_1 OK668727_1 OK668726_1 OK668725_1 OK668724_1 OK668731_1 OK668723_1 OK668722_1 OK668721_1 OK668762_1 OK668755_1 OK668754_1 OK668752_1 OK668761_1 OK668753_1 OK668720_1 OK668719_1 OK668718_1 OK668717_1 OK668716_1 OK668715_1 OK668714_1 OK668713_1 OK668730_1 OK668748_1 OK668825_1 OK668824_1]
[Hap_4: 1   OK668835_1]
[Hap_5: 1   OK668834_1]
[Hap_6: 18   OK668832_1 OK668747_1 OK668745_1 OK668744_1 OK668743_1 OK668741_1 OK668736_1 OK668756_1 OK668733_1 OK668734_1 OK668735_1 OK668732_1 OK668742_1 OK668740_1 OK668739_1 OK668738_1 OK668737_1 OK668746_1]
[Hap_7: 1   OK668831_1]
[Hap_8: 1   OK668830_1]
[Hap_9: 1   OK668828_1]
[Hap_10: 1   OK668819_1]
[Hap_11: 15   OK668818_1 OK668817_1 OK668812_1 OK668811_1 OK668810_1 OK668809_1 OK668774_1 OK668773_1 OK668772_1 OK668771_1 OK668770_1 OK668769_1 OK668768_1 OK668776_1 OK668775_1]
[Hap_12: 30   OK668814_1 OK668797_1 OK668796_1 OK668795_1 OK668791_1 OK668788_1 OK668787_1 OK668786_1 OK668785_1 OK668784_1 OK668783_1 OK668782_1 OK668801_1 OK668800_1 OK668790_1 OK668789_1 OK668781_1 OK668780_1 OK668804_1 OK668779_1 OK668778_1 OK668777_1 OK668798_1 OK668793_1 OK668792_1 OK668802_1 OK668799_1 OK668815_1 OK668803_1 OK668827_1]
[Hap_13: 1   OK668808_1]
[Hap_14: 1   OK668807_1]
[Hap_15: 2   OK668806_1 OK668805_1]
[Hap_16: 6   OK668763_1 OK668766_1 OK668765_1 OK668764_1 OK668767_1 OK668816_1]
[Hap_17: 1   OK668794_1]
[Hap_18: 1   OK668757_1]
[Hap_19: 1   OK668758_1]
[Hap_20: 1   OK668820_1]
[Hap_21: 1   OK668712_1]
[Hap_22: 1   OK668710_1]
[Hap_23: 1   OK668822_1]
[Hap_24: 1   OK668821_1]
[Hap_25: 1   OK668709_1]
[Hap_26: 1   OK668823_1]
[Hap_27: 1   OK668711_1]
[Hap_28: 1   OK668813_1]
[Hap_29: 1   OK668829_1]
[Hap_30: 1   OK668826_1]
[Hap_31: 1   OK668833_1]
