# Supplementary material for: Aedes koreicus, a vector on the rise: Pan-European genetic patterns, mitochondrial and draft genome sequencing
Source: PLoS One. 2022 Aug 1;17(8):e0269880. doi: 10.1371/journal.pone.0269880 (PMC9342712; doi:10.1371/journal.pone.0269880)
Supplement: S3 Appendix — (PDF) [file pone.0269880.s005.pdf]

S3 Appendix. Calculated Bayes Factors for specific dispersal routes of *Aedes koreicus* within the investigated European populations.

From	To	Bayes Factor
Maasmechelen<br>(Flandria, Belgium)	Arba<br>(Friuli Venzia Giulia, Italy)	31.221086662999646
Pécs<br>(Southern Transdanubia, Hungary)	Baon<br>(Veneto, Italy)	5.9431444460479055
Budapest<br>(Central-Hungary, Hungary)	Arba<br>(Friuli Venzia Giulia, Italy)	7.776197934391781
Budapest<br>(Central-Hungary, Hungary)	Castel Ivano<br>(Trentino-Alto Adige, Italy)	416.67334770003663
Villamontagna<br>(Trentino-Alto Adige, Italy)	Baone<br>(Veneto, Italy)	6.0750831084945105
Maniago<br>(Friuli Venzia Giulia, Italy)	Baone<br>(Veneto, Italy)	46.65826894455457
Pedavena<br>(Veneto, Italy)	Grigno<br>(Trentino-Alto Adige, Italy)	9.250757520355833
San Michele all'Adige<br>(Trentino-Alto Adige, Italy)	Grigno<br>(Trentino-Alto Adige, Italy)	4.673723620251378
San Michele all'Adige<br>(Trentino-Alto Adige, Italy)	Valli del Pasubio<br>(Veneto, Italy)	3.4058369274555003
Grigno<br>(Trentino-Alto Adige, Italy)	Castel Ivano<br>(Trentino-Alto Adige, Italy)	5502.518188580577
Arba<br>(Friuli Venzia Giulia, Italy)	Castel Ivano<br>(Trentino-Alto Adige, Italy)	5.583224432606171
Monteforte d'Alpone<br>(Veneto, Italy)	Baone<br>(Veneto, Italy)	6.300992241289952
Castel Ivano<br>(Trentino-Alto Adige, Italy)	Valli del Pasubio<br>(Veneto, Italy)	4.22011005416697
Castel Ivano<br>(Trentino-Alto Adige, Italy)	Baone<br>(Veneto, Italy)	51.15628021169566
Castel Ivano<br>(Trentino-Alto Adige, Italy)	Črniče<br>(Goriška, Slovenia)	8.022727454894238
Lovrenc na Dravskem polju<br>(Drava, Slovenia)	Baone<br>(Veneto, Italy)	8.78143589286883
Wiesbaden<br>(Hessen, Germany)	Valli del Pasubio<br>(Veneto, Italy)	1829.4080257224512
Wiesbaden<br>(Hessen, Germany)	Baone<br>(Veneto, Italy)	5.8260199803535215
Valli del Pasubio<br>(Veneto, Italy)	Baone<br>(Veneto, Italy)	30.35917468328573
Valli del Pasubio<br>(Veneto, Italy)	Vittorio Veneto<br>(Veneto, Italy)	8.299160285144584
